# Interrelationship Between Broadband NIRS Measurements of Cerebral Cytochrome C Oxidase and Systemic Changes Indicates Injury Severity in Neonatal Encephalopathy

**DOI:** 10.1007/978-3-319-38810-6_24

**Published:** 2016-05-02

**Authors:** Gemma Bale, Subhabrata Mitra, Isabel de Roever, Marcus Chan, Alexander Caicedo-Dorado, Judith Meek, Nicola Robertson, Ilias Tachtsidis

**Affiliations:** 10 Biomedical Optics Research Laboratory, University College London, London, UK; 11 Institute for Women’s Health, University College London, London, UK; 12 Department of Electrical Engineering ESTA, Katholieke Universiteit Leuven, Leuven, Belgium

**Keywords:** Near-infrared spectroscopy, Cytochrome c oxidase, Hypoxic ischaemic encephalopathy, Neonatal, Oxygen metabolism

## Abstract

Perinatal hypoxic ischaemic encephalopathy (HIE) is associated with severe neurodevelopmental problems and mortality. There is a clinical need for techniques to provide cotside assessment of the injury extent. This study aims to use non-invasive cerebral broadband near-infrared spectroscopy (NIRS) in combination with systemic physiology to assess the severity of HIE injury. Broadband NIRS is used to measure the changes in haemodynamics, oxygenation and the oxidation state of cytochrome c oxidase (oxCCO). We used canonical correlation analysis (CCA), a multivariate statistical technique, to measure the relationship between cerebral broadband NIRS measurements and systemic physiology. A strong relationship between the metabolic marker, oxCCO, and systemic changes indicated severe brain injury; if more than 60 % of the oxCCO signal could be explained by the systemic variations, then the neurodevelopmental outcome was poor. This boundary has high sensitivity and specificity (100 and 83 %, respectively). Broadband NIRS measured concentration changes of the oxidation state of cytochrome c oxidase has the potential to become a useful cotside tool for assessment of injury severity following hypoxic ischaemic brain injury.

## Introduction

Hypoxic ischaemic encephalopathy (HIE)Neonatal HIE resulting from hypoxic ischemic (HI) brain injury affects 1–2 live births per 1000 and is associated with severe neurodevelopmental problems and mortality [1]. Diminished supply of oxygen (hypoxia) and Hypoxic ischaemic encephalopathy (HIE) Hypoxic ischaemic encephalopathy (HIE) to the newborn brain in the perinatal period causes neuronal injury. HIE is an evolving process; after the initial period of energy failure during the injury, the cerebral metabolism recovers to normal for the first few hours of life but then can deteriorate, leading to a secondary energy failure (Secondary energy failure (SEF) Hypoxic ischaemic encephalopathy (HIE)) [2]. Strategies to treat HIE focus on preventing the cascade of events leading to SEF and, currently, therapeutic hypothermia (TH) has been the only successful treatment that is in routine clinical use [3]. The current gold standard assessment of the injury is the magnetic resonance spectroscopy (MRS) measured lactate to N acetyl aspartate ratio (Lac/NAA) which is the most sensitive predictor of outcome in the first 30 days of life [4]. However the magnetic resonance (MR) scan is generally performed at the end of the first week of life and a real-time, cotside measurement of cerebral metabolism that can assess the progression of cerebral injury would be helpful in the immediate stages following HI brain injury.

Our group has previously presented a broadband NIRS device called Near-infrared spectroscopy (NIRS) Hypoxic ischaemic encephalopathy (HIE) (CYtochrome Research Instrument and CYtochrome Research Instrument and appLication (CYRIL)) to monitor cerebral hemodynamics and metabolism in the newborn brain [5]. CYRIL measures the changes in concentration of oxygenated- and deoxygenated-haemoglobin (Δ[HbO_2_] and Δ[HHb], respectively) and the oxidation state of cytochrome c oxidase (Δ[oxCCO]). Cytochrome c oxidase (CCO) is the terminal electron acceptor in the electron transport chain in Cytochrome c oxidase (CCO). It is responsible for more than 95 % of Oxygen metabolism in the body as it is essential for the efficient generation of ATP [2]. The enzyme contains four redox centers, one of which—copper A (CuA)—has a broad absorption peak in the near-infrared (NIR) spectrum which changes depending on its redox state. As the total concentration of CCO is assumed constant, the changes in the NIRS-measured Oxidation state of cytochrome c oxidase (oxCCO) concentration are indicative of the Hypoxic ischaemic encephalopathy (HIE) redox state and, therefore, provide a representation of oxygen utilization in the tissue. Detection of CCO using Cytochrome c oxidase (CCO) CCO is more difficult than other chromophores as its in-vivo concentration is less than 10 % of that of haemoglobin and has a broad spectral signature. We use broadband (multi-wavelength) NIRS and the UCLn algorithm to accurately resolve spectral changes due to oxCCO without cross-talk from haemoglobin chromophores [6].

We hypothesize that the dynamic changes in the cerebral metabolism, as monitored by broadband NIRS measured Δ[oxCCO], in response to systemic changes will indicate brain injury severity. To test this, we use canonical correlation analysis (Hypoxic ischaemic encephalopathy (HIE)), a multivariate statistical technique that measures the relationship between two groups of variables [7]. CCA can be seen as an extension to normal correlation analysis where the relation between two multidimensional datasets (or group of signals) is analysed instead of two individual signals. In this way CCA can estimate the strength of the relationship between Canonical correlation analysis (CCA).

## Methods

Ethical approval for the Baby Brain Study at University College London Hospitals Trust (UCLH), London was obtained from the North West Research Ethics Centre (REC reference: 13/LO/0106). Term infants born at or transferred to UCLH for treatment of acute brain injury were eligible for investigation; only babies without congenital malformations were considered. Each subject was monitored continuously with EEG and treated with TH which was initiated within 6 h of birth; body temperature was lowered to 33.5 °C for 72 h.

Broadband Hypoxic ischaemic encephalopathy (HIE) were collected continuously over a period of 3 h on the third day of life during TH. We used a custom-built two-channel broadband NIRS system called CYRIL CYtochrome Research Instrument and appLication (CYRIL) that has been described previously [5]. All measurements were taken bilaterally on the forehead over the frontal lobe at a sampling frequency of 1 Hz. The changes in chromophore concentrations (Oxidation state of cytochrome c oxidase (oxCCO), HbO_2_ and HHb) were calculated from the measured changes in broadband near-infrared light attenuation using the modified Beer-Lambert law as applied with the UCLn algorithm across 136 wavelengths (770–906 nm) with a fixed differential pathlength factor of 4.99 and 2.5 cm optode separation.

Systemic data from the Intellivue Monitors (Philips Healthcare, UK) were collected using an application called ixTrend (ixellence GmbH, Germany). Signals recorded include oxygen saturation (SpO_2_) measured by pulse oximetry on the foot or hand, heart rate (HR) by electrocardiograph (ECG), respiratory rate (RR), mean arterial blood pressure (MABP) from an intra-arterial catheter, and transcutaneous carbon dioxide (PaCO_2_) and oxygen (PaO_2_) tension.

The babies spent 1 h in the MR scanner after rewarming, this occurred on average on day 7 of life (range: day 5–15). MR scans included a measurement of thalamic Lac/NAA with proton (^1^H) MRS; Lac/NAA score ≥ 0.3 has been shown to be associated with worse neurodevelopmental outcome.

Data analysis was carried out in MATLAB (Mathworks, USA) and Excel (Microsoft, USA). Systemic data were down-sampled and interpolated to the NIRS data timeframe (1 Hz). Both NIRS and systemic data were processed with an automatic wavelet de-noising function which reduces the high frequency noise but maintains the trend information. Artefacts in the NIRS signals from movement or changes in ambient lighting were removed using the method suggested by Scholkmann et al. [8] which is based on moving standard deviation and spline interpolation. This method also corrects shifts in the baseline due to artefact.

For the CCA analysis, the signals were grouped into ‘cerebral’ (HbO_2_, HHb and oxCCO) and ‘systemic’ sets (MABP, SpO_2_, HR, RR, PaCO_2_ and PaO_2_). The first step of CCA is to form linear combinations of the variables in each data set. Then the correlation between each group of variables is used to assess the dependency of the cerebral signals on the systemic signals. This gives a score that ranges from 0 to 1, where 0 indicates no dependency and 1 indicates total dependency. CCA results were compared with the severity of the injury which was assessed by MRS measured Lac/NAA on days 5–15 of life; the subjects were grouped into severe brain injury (Lac/NAA ≥ 0.3) and mild brain injury (Lac/NAA < 0.3). The median of the CCA scores for the dependency of each Canonical correlation analysis (CCA) on the systemic changes, also known as cross-loading coefficients, were found for each injury group as the data were not normally distributed. A Kruskal–Wallis Kruskal-Wallis test Hypoxic ischaemic encephalopathy (HIE) was used to assess the significance of the difference between the groups; the level of statistical significance was set at p < 0.05. The sensitivity and specificity of the CCA scores for injury classification are evaluated.

## Results

**Fig. 24.1:**
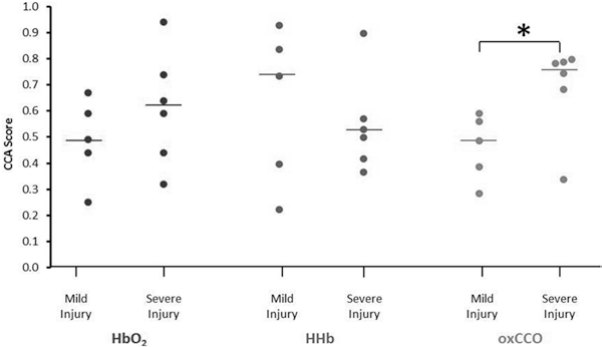
CCA Hypoxic ischaemic encephalopathy (HIE) for mild (n = 5) and severe (n = 6) groups for each Canonical correlation analysis (CCA). Median score for each group is displayed as a line. The Kruskal–Wallis test found a significant difference between the groups for oxCCO only: HbO_2_ p = 0.36, HHb p = 0.72 and oxCCO p = 0.04

Data were recorded from 11 subjects (6 female) with HIE: severe injury n = 6, mild injury n = 5. One of the severely injured neonates died a few months after birth. All infants were born at term gestation (mean 39 ± 1 weeks), and were of normal birth weight (mean 3.1 ± 0.6 kg). The CCA analysis showed that each of the cerebral signals could (in part) be explained by the systemic physiological variations; the Hypoxic ischaemic encephalopathy (HIE) had a mean CCA score of 0.55 ± 0.20 which means that 55 % of the HbO_2_ signal could be explained by systemic variations, the HHb and Oxidation state of cytochrome c oxidase (oxCCO) mean CCA scores were 0.59 ± 0.23 and 0.58 ± 0.18, respectively. The CCA scores for each cerebral variable were grouped into mild and severe injury categories and are shown in Fig. 24.1. There is no significant difference between the mild and severely injured groups in the dependency of either HbO_2_ or HHb on the systemic signals (p = 0.36 and 0.72, respectively). However, the infants with severe injury showed a significantly (p = 0.04) higher oxCCO dependency (median: 0.74) than those with mild injury (median: 0.49). The infants with severe injury were identified by an oxCCO CCA score ≥ 0.6 (sensitivity 100 %, specificity 83 %).

## Discussion

CCA showed that broadband NIRS measured cerebral signals could be explained, in part, by changes in the systemic physiology which is expected because the systemic physiology should influence the Hypoxic ischaemic encephalopathy (HIE). Although the relationship between changes in the haemoglobin signals (HbO_2_ and HHb) and systemic signals varied between the infants, their relationship was not indicative of injury as measured by Lac/NAA. The relationship between Δ[Oxidation state of cytochrome c oxidase (oxCCO)] and systemic physiology was able to predict injury; a high oxCCO dependency on the systemic signals indicated a negative outcome. This means that although the cerebral vascular response to systemic events was not significantly different between the mild and severe injuries, the cerebral metabolic response was. This could be explained by lower cellular energetics in the more injured brain which would mean that CCO has less capacity to buffer changes in oxygenation from systemic variations. It is possible that the differences in the metabolic responses are due to changes in the brain leading to, or after, Secondary energy failure (SEF) in the severely injured brain.

We have previously found a strong linear correlation between changes in Hypoxic ischaemic encephalopathy (HIE) and NIRS measured Near-infrared spectroscopy (NIRS) during systemic desaturation events in infants with severe HIE [5]. This relationship supports the findings in this study; in the more injured brain CCO is more readily affected by changes in oxygen delivery and therefore would be more disturbed by systemic variations.

There are many limitations to this study to discuss. Firstly, this is a small sample size. Secondly, the Lac/NAA is measured in the thalamus whereas the NIRS measurements are over the frontal lobe, and the Lac/NAA is only a surrogate for patient outcome; a neurodevelopmental outcome will be a more robust indicator. Finally, the Hypoxic ischaemic encephalopathy (HIE) technique assumes a linear and stationary relationship between all of the variables which may not be true in this complex system; in this study CCA examines the relationship over a long window of time (3 h) during which the relationship between the variables might change; in future, we could use temporal CCA to investigate the relationship between cerebral and systemic variables over time.

## Conclusions

We identified severe HIE from mild HIE with an early cotside biomarker of metabolism. A strong relationship between oxCCO and systemic physiology, assessed with CCA, on day 3 of life indicated severe injury. The broadband NIRS measured Oxidation state of cytochrome c oxidase (oxCCO) is more sensitive to brain injury than HbO_2_ or HHb.
